# Technetium-99m scan in the laparoscopic management of a misdiagnosed Meckel's diverticulum: a case report

**DOI:** 10.1186/1752-1947-3-6981

**Published:** 2009-04-29

**Authors:** Michael Pitiakoudis, George Vaos, Michael Kirmanidis, Stefanos Gardikis, Evanthia Tsalkidou, Constantinos Simopoulos

**Affiliations:** 1 2nd Department of Surgery, Alexandroupolis University Hospital, Democritus University of Thrace School of Medicine, 68100 Alexandroupolis, Greece; 2 Department of Pediatric Surgery, Alexandroupolis University Hospital, Democritus University of Thrace School of Medicine, 68100 Alexandroupolis, Greece

## Abstract

**Introduction:**

Although Meckel's diverticulum is the most common congenital abnormality of the gastrointestinal tract and modern imaging techniques are available, its diagnosis remains problematic.

**Case presentation:**

A technetium-99 scan was performed in an 18-year-old man with abdominal pain, vomiting and rectal bleeding to confirm the presence of a Meckel's diverticulum which was not diagnosed laparoscopically elsewhere. The technetium-99 scan was positive and a diagnostic laparoscopy was re-performed which revealed a Meckel's diverticulum that was subsequently resected.

**Conclusion:**

We suggest that a technetium-99m scan should be performed before laparoscopy in children and adolescents with suspected Meckel's diverticulum. A positive technetium-99m scan may significantly contribute to the laparoscopic definitive diagnosis and treatment of a bleeding Meckel's diverticulum. However, diagnostic laparoscopy should only be performed by experienced surgeons.

## Introduction

Meckel's diverticulum (MD) is the most common congenital abnormality of the gastrointestinal tract, occurring in 1% to 3% of the population [1]. It is the remnant of the omphalomesenteric duct (vitelline duct), which is normally obliterated by the fifth week of gestation and usually contains two types of heterotopic mucosal tissue: gastric and pancreatic [2]. Although only 4% of MDs become symptomatic, very often their first symptoms are associated with serious complications such as inflammation, perforation, bleeding, intussusception or intestinal obstruction [1]. If a complicated MD is suspected based on symptoms such as bleeding or abdominal pain, the technetium-99 scan (Tc-99m scan) is the examination that frequently leads to the diagnosis pre-operatively [1]. Since the symptoms are often non-specific and are attributed to other pathologies and investigation of the distal ileum is frequently not diagnostic, the majority of complicated MDs tend to be discovered incidentally during a surgical exploration of the abdomen [1]. In the case reported here, laparoscopy was used not only as a diagnostic tool, but also to treat the bleeding MD. The Tc-99m scan was helpful in the pre-operative diagnosis and laparoscopic confirmation of the MD.

## Case presentation

An 18-year-old man was referred to the accident and emergency department of our hospital with a 5-day history of abdominal discomfort, vomiting and fresh blood in his stools. The patient had passed bloody stools five times in the last 36 hours. The clinical evaluation revealed diffuse abdominal pain, localized mostly in the right lower quadrant with no signs of peritoneal irritation. The rectal examination was positive for blood. His vital signs were: blood pressure 110/70 mmHg, pulse rate 92/minute, respiratory rate 16/minute, and temperature 37.10 C. Physical examination of the other systems showed no abnormality. Laboratory tests returned the following values: white blood cell count 7.53 K/μL (Neut. 56.2%, Lymph. 34.7%, Mono. 6.6%, and Eos. 2.0%); red blood cell count, 3.94 M/μL; hematocrit, 29.3%; hemoglobin, 11.3 g/dL; platelets, 239 K/μL; C-reactive protein, 0.1 mg/dl; erythrocyte sedimentation rate, 1 mm/hour; and normal biochemical parameters and urinalysis. The patient had a long history of recurrent abdominal pain. From the age of 12, he started occasionally having mild abdominal pain located mostly in the right lower quadrant and radiating into the back. He was not passing bloody stools. Three years previously, an ultrasound of the upper and lower abdomen was performed which revealed findings of ileitis. Therefore a computed tomography (CT)-scan of the abdomen with contrast was obtained which showed a thickened wall of the terminal ileum and a small amount of fluid at the lower limit of the cecum, inside the small pelvis. The possibility of the presence of an inflammatory disease of the bowel was considered and a colonoscopy with multiple biopsies was performed. The colonoscopy showed no macroscopic abnormalities of the large-bowel mucosa and the terminal ileum (15 cm proximal to the ileocecal valve). However, histological findings were compatible with Crohn's disease. Although the patient received mesalazine, his clinical condition deteriorated and therefore a more detailed evaluation was performed including video capsule endoscopy, CT enteroclysis with barium meal and magnetic resonance imaging (MRI) of the abdomen. Nevertheless, these investigations showed no abnormality. An exploratory laparoscopy was undertaken for suspected MD without discovering any findings which could explain the patient's symptomatology and an incidental appendicectomy was performed.

**Figure 1:**
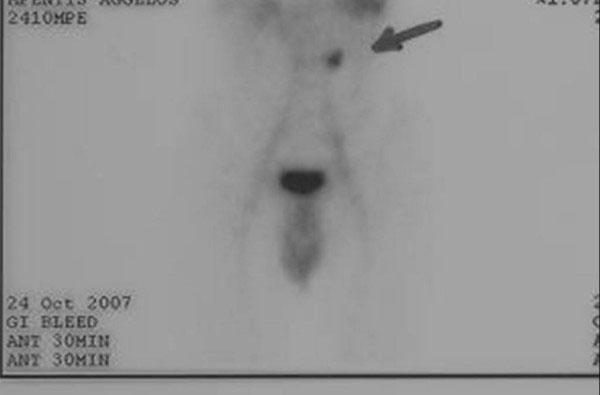
**Positive result of technetium-99m imaging**.

The patient was admitted to our hospital for further investigation. A Tc-99 m scan was performed which revealed an accumulation of pertechnetate in the abdomen laterally to the bifurcation of the iliac artery (Figure 1). This finding was compatible with a MD and a second laparoscopic exploration was performed. Under general anesthesia, a 10-mm subumbilical port for the laparoscope was inserted by the Hassan technique and a pneumoperitoneum was created with carbon dioxide insufflation at a pressure of 12 mmHg. Two working ports (5-mm and 10-mm) were inserted into the lower abdomen to facilitate bowel examination. The general laparoscopic examination was negative for gross intra-abdominal lesions. Through inspection with a 0 grade laparoscopic optic fiber, the ileocecal junction was identified. A carefully step-wise inspection from the ileocecal junction proximally was accomplished. A MD was found, located 50 cm proximal to the ileocecal valve. After dissecting the mesenterium with Ligasure, the MD was resected by tangential excision using an Endo-Gia-stapler and it was removed using an Endocath. The histological examination of the resected specimen confirmed the presence of heterotopic gastric mucosa (HGM) and the complete resection. Recovery was uneventful and the patient was discharged on the fifth postoperative day. The patient remains asymptomatic 6 months after surgery.

## Discussion

According to a statement from the Mayo Clinic, "Meckel's diverticulum is frequently suspected, often looked, and seldom found". Therefore, the diagnosis of MD is difficult [3]. There are many diagnostic examinations that can be useful in the diagnosis of MD, but most of them have their own limitations [2]. Visceral angiography is an invasive procedure and because of that, it is rarely used to establish the diagnosis of MD. Occasionally, it can be useful when a MD is bleeding [3]. Dense capillary staining of the vitelline artery permits the imaging of MD in the absence of bleeding [4]. CT enteroclysis with barium can be a diagnostic option if an acute abdomen does not exist, because it prevents emergency surgery [5]. In our patient, the video capsule endoscopic study failed to reveal the presence of the MD. In contrast, Geier *et al.*[6] reported a case of a 17-year-old patient with profuse rectal bleeding where video capsule endoscopic study found the MD. However, they confirmed the capsule endoscopic findings with Tc-99m scan.

Tc-99m scan is a widely accepted imaging modality for the discovery of a MD, especially in children with lower gastrointestinal (GI) bleeding [7]. The affinity of Tc-99m for gastric mucosa renders this imaging study a very valuable diagnostic tool in the detection of HGM [7]. However, if HGM does not exist in the MD, the diagnosis is problematic [2]. Tc-99m scan in the diagnosis of MD has a sensitivity of 85% and a specificity of 95% in children, but in adults these values fall to 65% and 9%, respectively [3]. Causes of false positive results include intussusception, bowel inflammation, GI bleeding unrelated to HGM, ureteric obstruction, vascular lesions such as hemangiomas and arteriovenous malformations [7]. False-negative results can occur when HGM is very slight in the MD, if necrosis of MD has occurred, or if the diverticulum is missed because it is superimposed on the bladder [2].

Despite the availability of modern imaging techniques, diagnosis of MD is challenging. In a study of 17 patients, the diagnosis of MD was established in 8 (76%) patients with laparoscopy. Only 4 (50%) of these eight patients had positive Tc-99m scan findings [8]. In another study of 12 children who presented with rectal bleeding, laparoscopy was able to make a correct diagnosis of a MD in all these symptomatic patients [9]. Therefore, some authors suggest replacing the Tc-99m scan by diagnostic laparoscopy [9,10]. However, this may lead to misdiagnosis of a MD as occurred with our own patient. A Tc-99m scan performed before laparoscopy might have been useful in the case presented here. Laparoscopic examination has also been proposed for pediatric patients with obscure lower GI bleeding regardless of the results of the Tc-99m scan, since MD, although usually asymptomatic, comprises the most common cause of lower GI bleeding in pediatric and adolescent patients [11].

When the findings from the Tc-99m scan are uncertain for the diagnosis of a MD, a diagnostic laparoscopy should be performed. The safety and efficacy of diagnostic but also therapeutic laparoscopy are widely accepted [2]. A great variety of laparoscopic techniques have been used in the surgical treatment of MD [12]. Attwood *et al.*[13] performed laparoscopy-assisted extracorporeal Meckel's diverticulectomy for inflamed MD using an endoscopic mechanical stapler. Ng *et al.*[14] advocated segmental small bowel resection for bleeding MD [2]. In the laparoscopic procedure, the main argument is the possibility of incomplete resection of the ectopic tissue. The advancement of the laparoscopic stapler device and the increased experience with the laparoscopic technique have reduced the frequency of diverticulectomy-associated complications. However, the histological examination of the MD can ensure the diagnosis and that subsequent complete resection should be performed in the case of an incomplete resection [2].

## Conclusion

MD should be included in the differential diagnosis of recurrent abdominal pain or rectal bleeding, especially in children and adolescent patients. The most helpful non-invasive diagnostic tool in the diagnosis of a bleeding MD is the Tc-99m scan that should precede laparoscopy in patients with lower GI bleeding. In cases with suspected MD, a positive Tc-99m scan in combination with laparoscopy, performed by experienced surgeons, can definitively confirm the diagnosis and treat the patient.

## Abbreviations

CT: computed tomography; GI: gastrointestinal; HGM: heterotopic gastric mucosa; MD: Meckel's diverticulum; MRI: magnetic resonance imaging; Tc-99m scan: technetium-99 scan.

## Consent

Written informed consent was obtained from the patient for publication of this case report and any accompanying images. A copy of the written consent is available for review by the Editor-in-Chief of this journal.

## Competing interests

The authors declare that they have no competing interests.

## Authors' contributions

MP conceived the study, performed the laparoscopic operation, followed up the patient, and wrote the manuscript. GV designed the study, contributed to the writing of the manuscript and drafted the final manuscript. MK contributed to the writing of the manuscript and followed up the patient. SG helped with study design and contributed to the writing of the manuscript.

ET followed up the patient and reviewed the literature. CS helped with design of the study and contributed to the writing of the manuscript. All authors read and approved the final manuscript.
